# The cross-talk between the macro and micro-environment in precursor lesions of pancreatic cancer leads to new and promising circulating biomarkers

**DOI:** 10.1186/s13046-024-03117-5

**Published:** 2024-07-18

**Authors:** Carla Mottini, Francesca Romana Auciello, Isabella Manni, Christian Pilarsky, Damiano Caputo, Giulio Caracciolo, Alessandro Rossetta, Elena Di Gennaro, Alfredo Budillon, Giovanni Blandino, Maria Serena Roca, Giulia Piaggio

**Affiliations:** 1 Department of Research, Diagnosis and Innovative Technologies, UOSD SAFU, IRCCS Regina Elena National Cancer Institute, 00144 Rome, Italy; 2 UOC Translational Oncology Research, IRCSS Regina Elena National Cancer Institute, 00144 Rome, Italy; 3 Universitätsklinikum Erlangen Klinik Für Chirurgie, Erlangen, Germany; 4 Università Campus Bio-Medico Di Roma, Rome, Italy; 5 Dipartimento Di Medicina Molecolare Sapienza, Università Di Roma, Rome, Italy; 6 FLIM LABS Srl, Rome, Italy; 7 Experimental Pharmacology, Istituto Nazionale Tumori—IRCCS—Fondazione G. Pascale, Via M. Semmola, 80131 Naples, Italy; 8 Scientific Directorate, Istituto Nazionale Tumori—IRCCS—Fondazione G. Pascale, 80131 Naples, Italy

**Keywords:** Pancreatic cancer, Macro-environment, Micro-environment, Immune landscape, Circulating biomarkers

## Abstract

Pancreatic cancer (PC) is a clinically challenging tumor to combat due to its advanced stage at diagnosis as well as its resistance to currently available therapies. The absence of early symptoms and known detectable biomarkers renders this disease incredibly difficult to detect/manage. Recent advances in the understanding of PC biology have highlighted the importance of cancer-immune cell interactions, not only in the tumor micro-environment but also in distant systemic sites, like the bone marrow, spleen and circulating immune cells, the so-called macro-environment. The response of the macro-environment is emerging as a determining factor in tumor development by contributing to the formation of an increasingly immunogenic micro-environment promoting tumor homeostasis and progression. We will summarize the key events associated with the feedback loop between the tumor immune micro-environment (TIME) and the tumor immune macroenvironment (TIMaE) in pancreatic precancerous lesions along with how it regulates disease development and progression. In addition, liquid biopsy biomarkers capable of diagnosing PC at an early stage of onset will also be discussed. A clearer understanding of the early crosstalk between micro-environment and macro-environment could contribute to identifying new molecular therapeutic targets and biomarkers, consequently improving early PC diagnosis and treatment.

## Introduction

Among the different types of neoplastic diseases, pancreatic cancer (PC) has one of the deadliest outcomes carrying an overall 5-year survival rate of 12% [1]. Around 80% of patients with PC are diagnosed with locally advanced or metastatic disease without any current effective treatment options available [2]. To date, surgical resection remains the only option in early-stage patients, despite the fact the 5-year survival rate continues to remain low because of the high recurrence rate [3]. The outcome of patients with PC remains poor, mainly due to late-stage diagnosis, the absence of early and reliable diagnostic biomarkers and the lack of effective treatments.

Since the diagnosis and treatment of PC at its early stages is priority, identifying the distinctive features of the precursor lesion is crucial in treating patients before they develop invasive PC. The currently recognized pre-neoplastic lesions of PC are pancreatic intraepithelial neoplasms (PanIN), intraductal papillary mucinous neoplasms (IPMN), intraductal oncocytic papillary neoplasms, intraductal tubulo-papillary neoplasms and mucinous cystic neoplasms (MCN). Among them, PanIN, IPMN, and MCN are rather well characterized [4].

**Fig. 1:**
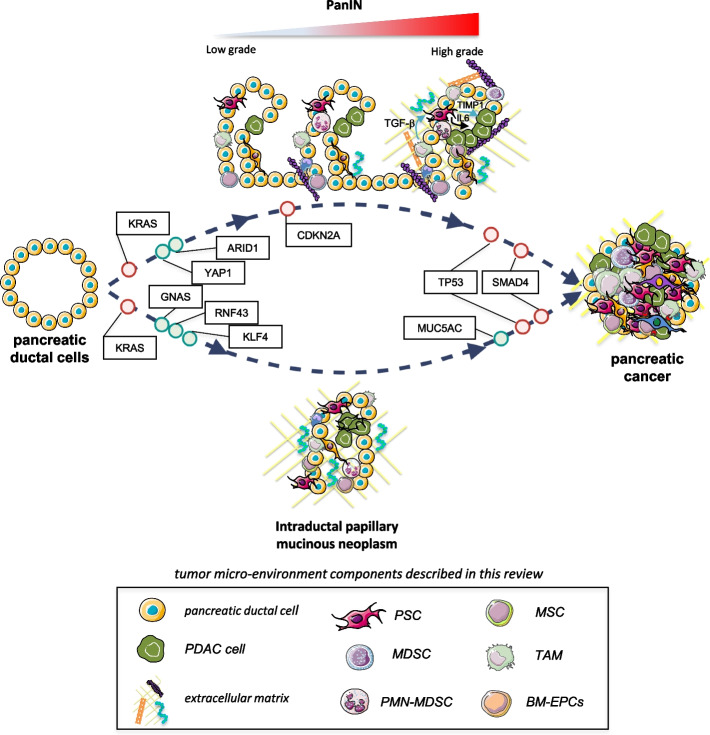
Evolution of tumor micro-environment composition from most common pancreatic cancer precursor lesions to PC considering mutational landscape. Progression from pancreatic cancer precursor lesions to PC is characterized by an high dynamism involving both multiple genetic mutations and heterogeneity of tumor micro-environment. In details, KRAS, CDKN2A, TP53 and SMAD are the key mutations in both PanINs and IPMNs. In PanIN, CDKNA and KRAS mutations are observed in the earliest stages, whereas TP53 and SMAD arise later. Other mutations cooperate to drive disease progress, such as YAP1 and ARID1, frequently observed in PanIN and MUCA5C, GNAS, RNF43 and KLF4, more selective for IPMN. The heterogeneity of tumor micro-environment is determined by a complex signaling networks between tumor, stromal and immune cells that leas to tumor progression of PanIN, along IPMN to cancer. Beyond cancer associated fibroblast (CAFs), pancreatic stellate cells (PSCs), and other stromal components, myeloid progenitors and haematopoietic stem cells are some of the main players involved in this cross-talk. As a result of cytokines and growth factors released by the tumor, these cells undergo an altered differentiation process, acquiring immunosuppressive properties capable of counteracting the anti-tumor immune response. In the figure are reported the main actor involved in the cross-talk between TIME and TIMaE, as listened below: pancreatic stellate cells (PSCs), myeloid derived suppressor cells (MDSCs), polymorphonucleated myeloid derived suppressor cells (PMN-MDSCs), mesenchymal stem cells (MSCs), tumor associated macrophages (TAMs) and BM-derived endothelial progenitor cells (BM-EPCs)

PanIN is the most prevalent pancreatic ductal adenocarcinoma (PDAC) precursor, and its classification is currently simplified into two PanIN levels: low-grade (PanIN 1 and 2) and high-grade (PanIN 3) [5]. Disease progression involves an accumulation of different genetic alterations, as shown in Fig. 1, where the most common early events in low-grade PanINs include mutation of *KRAS* gene, loss of *CDKN2A* oncosuppressor gene (coding for p16INK4A) and telomere shortening [6–9]. Loss of oncosuppressors *TP53* and *SMAD4* are late genetic events often detected when the high grade PanIN evolves into PC [6, 8–10].

Acinar-to-ductal metaplasia (ADM) of the pancreas is the process through which acinar cells differentiate into ductal-like cells. With oncogenic genetic insults and/or sustained environmental stress, in preclinical models, ADM may lead to PanIN [11]. The progression of ADM to PanIN is also supported by *YAP1* which plays a key role in PC and in the early stages of carcinogenesis [12]. Furthermore, *ARID1A* is required to maintain terminal differentiation of pancreatic acinar cells and its knockout accelerates both ADM development to PanINs and PanIN progression to more advanced stages by reducing KRAS-induced senescence [13–15].

IPMN rank among the most common pancreatic cystic tumours [16] and are a relevant research focus because they are clinically detectable precursor lesions of PDAC [17]. Although they exhibit distinct histological features from PanIN lesions, these precursor lesions share some common genetic mutations including point mutation in *KRAS*, and inactivating mutations in *TP53*, *CDKN2A* and *SMAD4* [18, 19]. Common drivers additionally found in IPMN include point mutations in the *GNAS* gene and inactivating mutations in *RNF43* and *KLF4* [20]**.**

PC is characterized by a highly dynamic and heterogeneous tumor microenvironment (TME), consisting of tumor cells, immune cells and fibroblasts, as well as acellular constituents, which have been extensively described elsewhere [21–26]. Recent studies suggest that there is a link between genetic alterations in tumor cells and the induction of an altered TME, which subsequently affects tumor development [27]. Activated stromal cells as well as immune cells have already been found in the stroma of PanIN lesions [28, 29], underlining how the presence of these cell types may be crucial for the progression of early lesions to PC. Immune infiltration occurs in PanIN and IPMN [30, 31], thus facilitating immune evasion as well as contributing to tumor progression, metastasis and PC drug resistance [26].

This review focuses on the surrounding systemic immune compartments, which, from now on, will be referred to as the Tumor Immune Macro-Environment (TIMaE) and will discuss how it interacts with the immune compartment of the TME, known as the Tumor Immune Micro Environment (TIME) [32].

First, we will give an overview of the most recently introduced concept of TIMaE. Then, we will address the different molecules (i.e. cytokines and growth factors) that mediate the feedback loop of signals between TIME and TIMaE, which allow the development of PC right from its early stages. Finally, we will give an overview on the existing PC biomarkers and early-stage clinical trials, while speculating on the possibility that cytokines and other molecules which mediate the cross talk between TIMaE and TIME could become new potential biomarkers in the near future.

### The emerging role of the TIMaE in PC

While most of the studies in the field had their focus on the understanding of local immune responses, the field of cancer immunology has now expanded its horizons towards studying the complex, active and constant interplay between TIME and TIMaE [33–36].

**Fig. 2:**
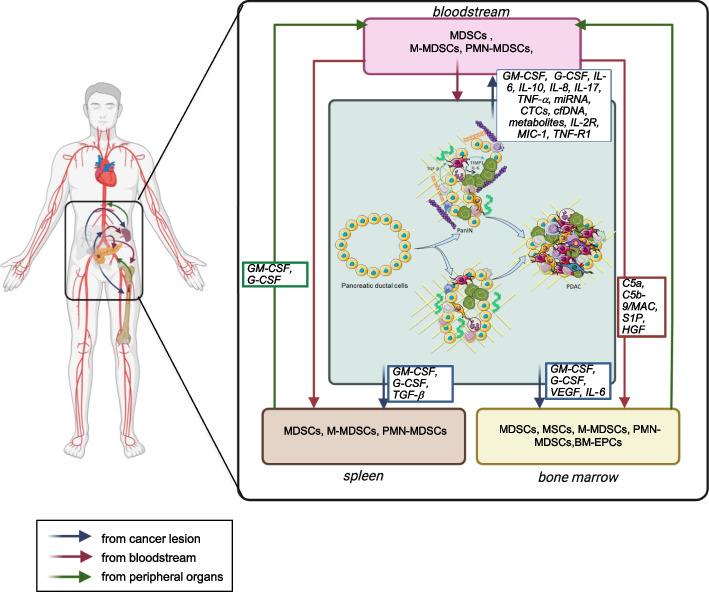
The sinergic crosstalk between macro and microenvironment in pancreatic cancer. Mobilization of hematopoietic stem and progenitor cells from the bone marrow and spleen to tumor site represents a key immune regulatory event in pancreatic cancer and early stages of disease. Growth factors released by pancreatic tumor cells (VEGF, Angiopoietin-1, IL-6, IL-8) promote a dysregulated hematopoiesis leading to mobilization of stem cells (BM-derived endothelial progenitor cells (BM-EPCs), mesenchymal stem cells (MSCs), in tumor microenvironment and their differentiation into immunosoppressive cells populations. Complement component C5a and C5b-9/MAC, sphingosine 1-phosphate (S1F) and Hepatocyte growth factor (HGF) are highly correlated with MSC mobilization. MDSC mobilization from bone marrow and spleen occur at early stage of the disease and is promoted by several tumor factors such as IL-6, CCL-2, IL-10, GM-CSF, G-CSF, SCF and CCL2. CCR2 mediated signaling drive monocyte mobilization from BM and differentiation in TAM. Pancreatic stellate cells (PCS) differentiate from BM progenitor cells and exert a key funtional role in early stage and cancer progression. Some of the factors that are engaged in the tumour-bone marrow cross-talk are currently recognised as diagnostic markers in pancreatic cancer. These include IL-6, VEGF, GM-CSF, G-CSF and TNF-a. In addition to date several inflammatory citokines (IL-17, IL-2R, IL-8, 1L-10) metabolites, miRNA, exosomes, cfDNA and CTCs rapresenting to date the promising blood biomarkers in early pancreatic cancer lesions and tumor

Although an official definition of TIMaE is yet to be defined, it is commonly accepted that it includes the following components: bone marrow, spleen, lymphatic vessels and the blood stream, along with inflammatory and hormonal factors [37] (Fig. 2).

The interaction between cancer cells, the microenvironment and these distant organs/systems is promoted by the tumor-associated neo-vasculature which mediates the release of several factors from the tumor itself that shape the host environment [37]. This crosstalk is vital for promoting tumor growth, proliferation, angiogenesis, invasion, and metastasis [38]. Among the most critical factors driving tumor progression is the evasion of the immune system, facilitated by immunosuppressive cells that can thwart the body’s immune response [39]. Numerous studies have shown that tumor cells have the ability to render immune cells anergic or even switch their function from anti-tumor to pro-tumor [34, 39]. Additionally, tumor cells can induce immunosuppression at distant sites from the tumor by releasing various factors and cytokines. These substances lead to a reprogramming of immune cell populations and their functions, particularly in lymphoid organs such as the bone marrow, spleen, and draining lymph nodes [35, 39]. Systemic alterations include changes in the functioning of the bone marrow and spleen, where especially myelopoiesis is heavily altered in the presence of a tumor. Briefly, immature myeloid cells, which acquire immunosuppressive activity due to the failure of their normal differentiation, move from the bone marrow into the bloodstream, thus regulating anti-tumor immunity and promoting cancer cell survival [36, 40]. The same phenomenon has often been observed during splenic myelopoiesis in mouse models [35, 41]. In addition to the aberrant hematopoiesis of monocytic and neutrophil cells, it is now known that CD8^+^ and CD4^+^ T-cell populations decrease along with dendritic cells, thus affecting the anti-tumor immune response [38, 42]. Taken together, these observations suggest a key role of peripheral immunity in suppressing antitumor activity, both by increasing the proportion of immunosuppressive lineages as well as by disrupting important mediators of antitumor immune responses. Preclinical and clinical models of PC demonstrate an altered myelopoiesis with an extensive mobilization of bone marrow-derived cells implicated in the generation of immunosuppressive subsets, such as myeloid-derived suppressor cells (MDSCs), capable of counteracting the antitumor response in the TIME [43, 44]. Moreover, splenic enlargement is evident due to the increased proportion of immunosuppressive CD15^+^ MDSCs in the spleen [45]. Both MDSCs and tumor associated macrophages (TAMs) infiltrate human tumors, and the intra-tumoral density of both cell types correlating with poor survival [46, 47].

These data strongly support a scenario in which myeloid cell-derived immunosuppression, along with the recruitment of immunosuppressive cells represent key immune regulatory pathways in PC even at early tumor stages.

### Impact of the TIME-TIMaE-TIME feedback loop on early lesions and progression of PC

Recently, a new key role has emerged for the different tumor- and TIME-derived factors in tumor development and progression. Their impact on TIMaE, involving myelopoiesis’ promotion along with unaltered differentiation of progenitor cells in the bone marrow (BM), spleen and peripheral blood [36, 48–52] has been underlined.^.^ This altered systemic differentiation results in the expansion and recruitment of myeloid lines in the TIME and are strongly correlated with disease progression, metastasis and poor prognosis [49, 50, 53–55]. To date, numerous lines of evidence show the mobilization of bone marrow stem cells in PC and their role in the modulation of tumor growth [56–58]. Peripheral blood samples from PC patients were analyzed, revealing a pronounced mobilization of stem cells, in particular very small embryonic stem cells (VSEL) and mesenchymal stem cells (MSC) [58]. It was observed that high levels of VEGF released by tumor cells enhance the migration of MSCs into tumor endothelial vessels that in turn release VEGF, thus supporting angiogenesis [59].

In addition to neovascularisation, MSCs mediate tumor invasion and progression by regulating the epithelial-mesenchymal transition [56, 57, 60, 61, 58, 59]. Coupled with VEGF, Antoon et al. showed that IL-6, and IL-8 released by MSCs create a pro-tumorigenic environment and induce STAT-3 phosphorylation in tumor cells. By blocking IL-6 as the main downstream effector, cell proliferation and tumor growth was reversed in vivo. Furthermore, in vivo co-injection of MSCs and tumor cells resulted in a 90% increase in tumor incidence compared to 50% in tumors alone [48].

BM-derived endothelial progenitor cells (BM-EPCs) are a group of stem cells that respond to signals from tumor cells, migrating to the tumor sites and promote neovascularisation and tumor growth [62]. Research in the context of PC has revealed the mobilization of BM-EPCs during the growth of PC [63]. Both in vivo and in vitro experiments have shown a strong correlation between the levels of BM-EPCs in the bone marrow, blood and tumor tissue and disease progression [63]. Furthermore, high levels of circulating BM-EPCs are positively associated with disease stage but negatively correlated with overall patient survival [64]. Another subset of these cells, CD34^+^ progenitor cells, represents endothelial progenitor cells, that, in response to certain pro-angiogenic tumor factors, migrate into TIME, differentiate into mature endothelial cells and promote angiogenesis [65]*. *In vitro experiments revealed that these cells migrate to PC sites in response to high levels of pro-angiogenic factors such as VEGF and Angiopoietin-1 secreted by tumor cells [66].

Apart from the above mentioned, a number of different cells, compose and interact with the cellular and acellular components in the TIME, thus modulating tumor progression. In this section, we will not only elucidate their origin but also describe how they are recruited from peripheral systemic organs by the tumor itself, already at its early stages. This will highlight the importance of a bidirectional communication between tumor tissues, lymphoid organs and tumor tissues to enhance the formation of TIME again, thus supporting tumor growth. In this context, myeloid-derived suppressor cells (MDSCs), a heterogeneous population of immature cells of myeloid origin, seem to have a pivotal role [67, 68]. Several lines of evidence show that tumor cells and tumor-associated stromal cells release inflammatory and tumorigenic factors that promote myelopoiesis, differentiation, expansion and homing of MDSCs, from the BM and spleen [49–52, 55, 69–71]. These cells represent the main immunosuppressive population in tumors that are able to hinder both innate and adaptive immune responses, thus promoting tumor progression and inducing resistance to therapies [67, 68, 72]. In the context of PC, preclinical and clinical studies demonstrated an altered myelopoiesis, driven by the release of pro-inflammatory cytokines and tumorigenic factors (IL-6, CCL-2, IL-10, GM-CSF, G-CSF, SCF and CCL2) that promote the mobilization of MDSCs from BM to TIME and a subsequent differentiation towards an immunosuppressive phenotype [43]. MDSCs begin to infiltrate during the early stages of the disease and steadily increase in number with tumor progression [40, 73–75]. In a study characterizing immunosuppressive cells in the human and murine PC model, an RNA sequencing analysis identified different populations of polymorphonuclear cells (PMNs) by distinguishing classical non-protumoral PMNs and PMN-MDSCs with potent immunosuppressive activity. These cell populations were also prevalent in PanINs, demonstrating that the immunosuppressive activity of PMN-MDSCs occurs very early in tumor development [74, 75]. Following depletion of CD11^+^/Gr-1^+^ myeloid progenitor cells in preclinical PC models, tissue repair was observed in PanINs despite oncogenic KRAS expression, indicating that MDSCs are required for PanIN lesion formation. However, KRAS is a known regulator of MDSC recruitment also in early PC lesions, mediating the release of GM-CSF by tumor cells [76].

The increase of MDSCs in the TIME of PC is also associated with impaired splenic erythropoiesis. Recently, greater attention has been paid to extramedullary hematopoiesis due to its significant involvement in generating a highly immunosuppressive TIME. Similar to what occurs in the BM, tumor secreted factors drive the expansion and differentiation of splenic stem and progenitor cells into cell populations that have an MDSCs-like genetic signature, thus revealing the role of cancer in the re-education of the splenic niche during the development of immunosuppressive cells [66, 67, 77]. A recent study in preclinical models of PC showed that tumor development is accompanied by splenomegaly and expansion of PMN-MDSC cells at both the systemic, tumor and splenic levels [78]. Furthermore, PMN-MDSC levels in the spleen and blood were closely correlated with serum levels of GM-CSF [78]. However, other studies using conditioned media of splenic cells, derived from murine tumor cells, showed a significant enrichment of GM-CSF [79]. The mechanism of extramedullary erythropoiesis has also been documented in pre-invasive PC lesions. The earliest evidence of immune suppressive elements in the early stages of PC tumourigenesis in the splenic environment was observed in mouse models with constitutively activated KRAS [80]. An expansion and accumulation of MDSC cells was observed in the spleen when the mice were already bearing PanINs. Interestingly, MDSC numbers increased in the spleen along with disease progression [80]. Further characterizations in preclinical models identified tumor GM-CSF as the main promoter of the splenic increase of MDSCs in PanINs [76, 81]. Furthermore, MDSCs recruited from an orthotopic pancreatic tumor mouse model able to recapitulate PanIN lesions, when grown with splenic CD3^+^ T cells, demonstrated a clear suppression of lymphocyte proliferation, underlining the key role of these cells in influencing splenic hematopoietic processes [76, 81]. A very recent study focusing on the metabolic enzymes of PC showed that increased expression of CD73 nucleotide is crucial for the formation of PanIN lesions, and is highly associated with the impairment of splenic CD8^+^ cells and increased circulating MDSCs, thus contributing to the formation of an immunosuppressive environment as well as disease progression [82].

Another myeloid-derived cellular component that is crucial in the context of PC TIME, is represented by tumor-associated macrophages (TAMs). These cells represent key components in the promotion of cancer stemness, angiogenesis, immune suppression, metastasis formation and development of therapeutic resistance [83–85]. TAMs are highly plastic cells and, in response to stimulatory signals from TIME, they can either acquire a proinflammatory phenotype that exerts anti-tumor activities (M1 phenotype) or an anti-inflammatory phenotype that promotes tumor development (M2 phenotype) [86]. In PC, TAMs play a role in all stages of the disease, from the development of ADM to the formation of precancerous PanINs and IPMN lesions, to adenocarcinoma progression and metastasis [87–89, 31]. Sanford et al. show that these cells derive from the differentiation of monocytes mobilized from the BM through CCR2-mediated signaling. In particular, a high prevalence of inflammatory monocytes was observed in the blood of patients with PC while analysis of the BM revealed a concomitant reduction in them. This relationship highly correlates with poor patient survival, which supports a prognostic role of the inflammatory monocyte balance in blood and BM. Furthermore, mouse models treated with a CCR2 antagonist showed a reduction in circulating monocytes, reduced tumor growth and fewer metastases, strongly pointing out the role of bone marrow cells in promoting PC [90]. In the very early stages of the disease, macrophages develop a pro-tumorigenic M2 phenotype and release inflammatory factors that promote neoplasia progression. They do this through the bidirectional communication between KRAS and the inflammatory factors released by tumor cells themselves. To date M2 TAMs represent the major macrophage component in PanINs where increasing fibrogenesis they promote the progression of pre-neoplastic lesions to PC [91–98].

Finally, we cannot conclude this section without mentioning pancreatic stellate cells (PSCs). These cells represent a key component in the formation of a highly desmoplastic and immunosuppressive pancreatic TIME, and there is evidence showing that PSCs can derive from the bone marrow progenitor cells [99, 100]. In healthy tissue, PSCs exist in a quiescent state and contribute to maintaining pancreatic stromal tissue architecture through the regulation of ECM homeostasis, amylase secretion, phagocytosis and innate immunity [101, 102]. Under the influence of oxidative stress, hypoxia, cytokines, growth factors released by tumor cells such as PDGF, TGF-β1 and specific signaling pathways (PI3-AKT, Wnt, JAK/STAT, and sonic hedgehog-Gli1), PSCs are recruited by the BM and from a quiescent state become activated [102]. The extensive and reciprocal cross-talk between tumor cells and activated PSC promotes an immunosoppressive TIME which progressively leads to tumor development [104]. Recruitment from lymphoid organs and activation of PCS is considered an early event in PC [103, 105, 106]. Studies on patients with pancreatitis have shown that BM-derived cells exhibit high levels of α-smooth muscle actin (α-SMA) expression, a marker of activated PSCs, suggesting the recruitment and differentiation of BM-derived cells into PCSs [107, 108]. Scarlett et al. reported an enrichment of BMSCs in the stroma proximal to PanIN lesions. These cells express markers typical of activated PSCs, thus confirming that PSCs derive from BM and are present in the early stages of PC development [103]. Other studies on PanIN cells isolated from mouse models of PC have demonstrated and confirmed the close relationship between PSCs activation and PanIN lesions progression [105, 109, 110]. In PanIN lesions, IL-6 was identified as a key secreted factor activating PSCs and promoting invasiveness [105].

In summary, as illustrated in Fig. 2, PC development is determined by a complex signaling network between tumor cells and components of TIME in advance PC and early lesions. However, some key cellular players in TIME have also an impact on peripheral lymphoid organs demonstrating the importance of the TIMaE in promoting a pro-tumoral and highly immunosuppressive environment. This provides a new insight on the interactions that characterize the tumor, focusing not only on the local micro-environment but also on the systemic macro-environment. Furthermore, since the crosstalk between TIME and TIMaE already occurs at early stages of the disease, its characterization will allow a deeper understanding of the mechanisms mediating the first steps of PC onset.

### TIME-TIMaE cross-talk fuels circulating diagnostic biomarkers for early detection of PC: preclinical and clinical evidence

Early diagnosis has become essential for patients with PC. However, the current available screening procedures are unable to detect PC in the early stages [111]. In this context, blood is easily accessible and relatively stable, making serum and/or plasma ideal specimens to discover biomarkers [112]. Technological advances in the last decade have provided more opportunities for discovering circulating biomarkers based on “omics” analyses, including methods focused on proteins, nucleic acids, circulating tumor cells (CTCs), and exosomes [112]. All these biomarkers gain greater scientific relevance when held responsible for the dynamic signaling implemented by early stage tumors that progress towards more aggressive forms.

One of the main sources of biomarkers resulting from an intense cross-talk between TIME and TIMaE is given by the inflammatory processes that are triggered and are responsible for tumor progression, as previously described. Indeed, various inflammatory factors, including cytokines, chemokines, and growth factors, have been shown to play important roles in tumorigenesis and can be considered excellent circulating biomarkers.

Cytokines play a key role in mediating the immune response to inflammatory or infectious stimuli [113]. In tumors, cytokines function by acting in either an autocrine or paracrine manner, as described in this review. They can infact stimulate growth of the tumor and the stromal cells that produce them, or promote recruitment, expansion and differentiation of immunosuppressive cells [114].

Cytokines detected in the bloodstream have recently emerged as markers of interest in clinical oncology research, particularly in predicting tumor prognosis and driving treatment choices [115]. PC patients often have inflammatory cytokines in the peripheral bloodstream acting as essential mediators in promoting an immunosuppressive environment within the TIME, also serving as predictive markers of disease.

In the context of potential biomarkers in PC, we would like to mention Th17 cells, a TIME-associated cellular component of PC that play a role in inflammation-induced tumorigenesis [116]. In a murine model, where knock-in of Kras in ductal acinar cells spontaneously induced PanIN formation, it has been shown that the stroma adjacent to areas of ADM as well as in PanIN is rich in Th17. These cells release IL-17, which, via the interaction with the highly expressed IL17ra receptor, accelerates the development and progression of PanIN [116]. However, Th17 cells in tumor tissue and serum levels of cytokines released by Th17 cells (IL-17 and IL-23) are present in more advanced stages of disease [117]. Moreover, it has been demonstrated in vitro that the endothelial CD34 + progenitor cells migrate to PC sites in response to high levels of pro-angiogenic factors such as VEGF and Angiopoietin-1, secreted by tumor cells. These cells also play a vital role in directing the differentiation of CD34 + cells into mature endothelial cells, capable of promoting angiogenesis [66].

A key role in the production of cytokines, considered potential circulating biomarkers, has also been attributed to PSCs [102, 103]. Indeed, once activated, PSCs secrete a large number of inflammatory signals that can promote angiogenesis, proliferation and migration of tumor cells, including IL-1, IL-6, IL-8, IL-10, VEGF, PDGF, FGF, type I collagen and CXCL12 [118, 119]. In response, tumor cells secrete cytokines such as IL-1, IL-6 and TNF-α and growth factors including TGF-β1 and platelet-derived growth factor BB (PDGF-BB).

These observations identify these cell subpopulations and their cytokines and growth factors as possible markers of disease and may have predictive roles in PC progression.

Several studies focusing on circulating inflammatory cytokines in IPMN have established their role as potential biomarkers for the diagnosis of this type of preneoplastic lesion. A comparison of circulating levels between patients with PC, IPMN and healthy subjects revealed a significant increase in serum levels of TNF-R1 in both PC and IPMN patients [120]. In Costa—Silva et al., the upregulation of exosomal macrophage migration inhibitory factor (MIF), an inflammatory cytokine involved in pre-metastatic liver niche formation and metastasis, was described. It is markedly higher in the exosomes of PC stage I patients, but interestingly, plasma exosomes containing MIF have been observed in the bloodstream of mice with PanIN pre-tumor pancreatic lesions. This evidence demonstrates the prognostic potential of metastatic risk of MIF in patients with pre-neoplastic lesions [121]. Finally, a recent prospective study of patients with IPMN examining circulating cytokine levels showed higher levels of TNF-α, IL-2R, IL-6 and IL-8 in patients with malignant IPMN [122].

In this field, there are ongoing clinical investigations focusing on sCD58 and TGF-β1, alone or in combination with carbohydrate antigen 19–9 (CA19-9), in serum samples from healthy individuals, along with those with pancreatitis, early lesions, and PC [123] (NCT05500027). Worth noting are also the results of the study of the CXCR2 Ligands/CXCR2 biological axis in the blood, tissue, and cystic fluid samples from healthy individuals, as well as in those with chronic pancreatitis, and PC (NCT00851955) (Table 1).

**Table 1:** Clinical trials evaluating circulating biomarkers in pancreatic cancer

Circulating Biomarkers								
Classification	Description	Type of sample	Conditions	Enrollment	Status	Trail number	Results/Trial titles	PMID
Arise from TIME-TIMaE cross-talk	CXCR2 Ligands/CXCR2 Biological Axis Amphoteric regulatory protein (AREG) Metabolome, metabolomic fingerprint Metabolomic fingerprint	blood| tissue| cystic fluid serum blood blood	healthy|Chronic pancreatitis|PC healthy|PC pancreatitis| cystic fibrosis| PC withPancreatic Exocrine Insufficiency high risk|PC	200 600 150 2522	COMPLETED UNKNOWN RECRUITING RECRUITING	NCT00851955 NCT04549064 NCT05980221 NCT04164602	no results no results no results no results	PMID: 33,444,177
Behind the TIME TIMaE cross-talk	sTRA and CA19-9 expression Elastase 1 sCD58 and TGFb1, alone or in combination with CA19-9 a consensus signature consisting of 29 biomarkers ctDNA for the diagnosis of early stage PC methylation-specific PCR of a 28 gene panel methylated DNA marker cfDNA methylation cfDNA methylation, serum protein markers, blood miRNA markers cfDNA methylation miRNA profile CIRcular and Non-coding RNAs microRNA-25 Circulating Extracellular Exosomal Small RNA	plasma serum serum serum blood|cyst fluid plasma blood| stool| pancreas cyst fluid| pancreas juice blood blood blood blood blood serum blood	healthy|PC healthy|high risk PDAC|PDAC healthy|pancreatitis|early lesions|PDAC healthy|early lesions|PDAC healthy|pancreatitis|early lesions|PDAC healthy|pancreatitis|PDAC healthy| early lesions healthy| early lesions|PC early lesions |PC healthy|high risk| early lesions|PC healthy| early lesions|PC healthy|pancreatitis|early lesions|PDAC healthy| early lesions|PC healthy| early lesions|PC	300 2100 2000 5000 750 330 800 276 450 7062 200 186 750 102	RECRUITING RECRUITING RECRUITING RECRUITING RECRUITING UNKNOWN RECRUITING NOT_YET_RECRUITING RECRUITING RECRUITING RECRUITING UNKNOWN NCT03432624 RECRUITING	NCT04143152 NCT06041009 NCT05500027 NCT03311776 NCT03334708 NCT02079363 NCT03855800 NCT06166147 NCT05495685 NCT05556603 NCT04406831 NCT04584996 NCT03432624 NCT04636788	sTRA is a validated serological biomarker of PDAC that yields improved performance over CA19-9 no results no results a biomarker signature was created, discriminating samples derived from patients with stage I and II from those from controls with a receiver operating characteristic area under the curve value of 0.96. no results A diagnostic prediction model (age > 65, BMP3, RASSF1A, BNC1, MESTv2, TFPI2, AP C, SFRP1 and SFRP2) no results no results no results no results no results no results no results no results	PMID: 30,617,132 PMID: 34,168,659 PMID: 30,106,639

As previously described, PC cells live in a harsh extracellular environment characterized by hypoxia, considerable desmoplasia and hypovascularization, rendering cancer cells more addicted to metabolic rewiring, or metabolic reprogramming in order to facilitate survival under these conditions. Recent technological advances have attracted more attention and interest in cancer-associated metabolic abnormalities [124]. Mayerle and colleagues showed that 9 serum metabolites (histidine, proline, sphingomyelin d18:2, sphingomyelin d17:1, phosphatidylcholine, isocitrate, sphingosine-1-phosphate, pyruvate, and ceramide), combined with CA 19–9, were able to distinguish between PC and chronic pancreatitis [125]. Interestingly, elevated plasma levels of branched-chain amino acids are an early event in PC development (when disease is still occult) and, at the time of diagnosis, are predictive of future tissue wasting [124]. Recently, it was possible to evaluate the combination of the three metabolites CA 19.9, TIMP1 and LRG1 in the detection of early stage PC through applying a metabolomic approach in plasma samples obtained from patients harboring non cancerous IPMN and IPMN patients with an associated invasive ductal adenocarcinoma [126]. Moreover, it has been proposed that the glycolytic enzyme and plasminogen receptor alpha-enolase (ENO1) as well as the transcription factor far upstream element-binding protein 1 (FUBP1) were upregulated in PC patients, leading to the production of autoantibodies (aAb) that discriminate healthy subjects from PC patients. It was highlighted that different levels of circulating aAb to ENO1 and FUBP1 could predict a poorer outcome [127].

These findings contribute to the delineation of blood metabolomic fingerprinting in individuals afflicted with pancreatitis, cystic fibrosis, and PC. In this context, two ongoing clinical trials (NCT05980221, NCT04164602) are actively recruiting participants, and we are very eagerly awaiting the results (Table 1).

### Circulating biomarkers behind the TIME TIMaE crosstalk

In addition to molecules exchanging signals between the organ and the periphery, many other biomarkers for PC have been described over the years. In this last section we would like to describe the clinical and preclinical studies that allowed the validation of traditional and new biomarkers for PC. Although these biomarkers are not related to host-periphery crosstalk, once combined with TIME-TIMaE crosstalk mediating molecules, could be considered an invaluable tool for diagnostics and clinical evaluation of PC patients, already at early stages. The most frequently used and most widely validated biomarker is CA 19–9, but, due to its low specificity and high false positive and negative rate, CA 19–9 is not sufficiently reliable as a diagnostic marker in clinical practice [128]. For this reason, some studies have focused on the combined detection of CA19-9 together with other tumor markers such as carcino-embryonic antigen (CEA), carbohydrate antigen 125 (CA125), carbohydrate antigen 242 (CA242), and on novel serum biomarkers [129], microRNAs [130] or new imaging techniques such as endoscopic retrograde cholangio pancreatography and endoscopic ultrasonography [131, 132]. In general, panels combining CA19-9 with other novel biomarkers may represent an ideal strategy to improve the sensitivity and specificity of CA19-9 in detecting PC.

Alongside studies aiming to strengthen and validate traditional biomarkers, there are also some clinical trials that are trying to identify new biomarkers (i.e. trial ID NCT06041009 and NCT04549064), and some of the newly proposed molecules even showed superior performance over CA19-9 (trial ID: NCT04143152) [133]. Among the studies that aim to evaluate circulating proteins the most successful clinical trial was the one that presented a consensus signature comprising of 29 biomarkers capable of discriminating between healthy individuals and those with PC in stages I and II [134] (Table I).

Although the emergence of cell-free DNA and tumour DNA as circulating biomarkers seem to represent a promising frontier in non-invasive diagnostics of PC, their effectiveness in pre-cancerous lesions remains controversial [135, 136]. Despite that, there is one trial (NCT03334708) with 750 enrolled participants which is currently assessing ctDNA for the diagnosis of early-stage PC. Apart from genetic abnormalities, an aberrant epigenetic modification, especially alterations in the methylation pattern, has also emerged as a potential biomarker for the early diagnosis of PC. Coupled with numerous targets (UCHL1, NPTX2, SARP2, CLDN5, FOXE1, CDH3) that display abnormal DNA methylation and are detectable in specific regions of cfDNA in PC, early studies are attempting to identify DNA methylated targets able to discriminate early lesions. For example, Liggett et al. proposed a 17-gene promoter panel that effectively differentiated chronic pancreatitis from controls (sensitivity = 81.7%, specificity = 78%) and PC patients (sensitivity = 91.2%, specificity = 90.8%). Coupled with that, several studies are actively recruiting patients to explore cfDNA methylation in different clinical conditions, including healthy individuals, those at high risk, and those with early PC lesions (NCT06166147, NCT05495685, NCT05556603). Methylation-specific PCR of a 28-gene panel was investigated in plasma samples from healthy individuals, those with pancreatitis, and in PC (NCT02079363). Moreover, Yi et al. reported that the promoter methylation status of *BNC1* and *ADAMTS1* in cfDNA is a promising biomarker for detecting early stage PC, showing a sensitivity of 81% and a specificity of 85% when evaluated together [137].

Other nucleic acids that are proving to be interesting biomarkers include circulating microRNA and lncRNA, which are tumor (and PC) specific and can be easily detected in the circulation of patients as potential diagnostic biomarkers of malignancy [138]. Even if the study of miRNA deregulation was well characterized as a very early event in the progression of PC and several miRNAs were identified to be involved in PanIN, IPMN and MCM progression, only a few of them were applied stepwise as biomarkers in plasma patients [139]. Interestingly, Lui J and colleagues evaluated the possibility of combining the plasma miRNA dosage with the early detection of CA-19–9, and claimed that the combination of CA19-9, miR-16, and miR-196a in the plasma is more effective in distinguishing PC from non-PC patients (normal or chronic pancreatitis), especially in early tumor screening [130]. By analyzing the expression of miRNAs contained in EVs from the plasma of PC mouse models, it was possible to identify 11 differentially expressed miRNAs in PC, healthy mice and PanIN, thus discriminating PC in the precancerous phase [140].

In Xu et al., interestingly, plasma exosome miRNA profiling in PC and IPMN allowed to discriminate and identify significantly elevated exosomal levels of miRNA-1246 in IPMN patients [141]. Once again, a serum expression analysis in tumors, healthy and IPMN patients showed that serum levels of miR-191, exosomal miR-21 and exosomal miR-451a substantially increased in PC and IPMN patients. The levels of miR-451a correlated with the clinical features of IPMN, with higher values found in patients with cysts large in diameter. This evidence places miR-451a as a potential biomarker in the malignant progression of IPMN [142]. Furthermore, in a study carried out by Abue et al. on miRNA expression levels in plasma samples from IPMN, PC and healthy patients, they detected a high expression of miRNA-483-3p and a higher expression of miRNA 21 in PC patients. However, miRNA-21 expression in plasma samples was higher in IPMNs compared to healthy individuals, suggesting miRNA-21’s role in early stage PC [143]. Furthermore, miR-483-3p is upregulated in PanIN, and its expression levels correlate with the progression of PanIN. Notably, circulating levels of miR-483-3p are significantly elevated in the serum and serum exosomes of patients with PC, confirming the evidence demonstrated by Abue et al. Interestingly, serum levels of miR-483-3p were shown to be able to effectively differentiate between patients with early stage PC (≤ 2 cm) and healthy controls [139]. Finally, a targeted study of exosomal miRNA profiles in early lesions and cancer showed differential expression of miRNA-196b-3p and miRNA-204-3p between IPNM, MCN and PC. In particular, the expression of miR-204-3p was significantly higher in exosomes deriving from the serum of MCN patients compared to those of IPMN patients [144].

Interestingly, numerous ongoing clinical studies are dedicated to identifying novel circulating miRNAs or miRNA signatures with the potential to detect pancreatic disease at its earliest stages in patients. For instance, in the NCT03432624 and NCT04584996 clinical trials, efforts have been made to enhance PC diagnosis by integrating the detection of microRNAs, traditional tumor markers, and imaging techniques. NCT04406831 investigates the significance of certain blood born miRNAs for early PC diagnosis, treatment response prediction, and prognostic insights in patients [139]. Moreover, the NCT04636788 clinical trial is looking for circulating extracellular exosomal small RNA in blood samples from healthy individuals and those with early lesions to obtain more suitable results **(**Table 1**)**.

In conclusion, our comprehensive analysis of diverse biomarkers, underscore the potential for improving early detection strategies for PC. Further research and consolidation of results from ongoing trials will contribute to refining and validating these promising biomarkers.

## Conclusions

Early diagnosis of PC is still an Achilles heel in oncological research, but recent progress in biomarker research shows promise in improving diagnostic accuracy. The interplay between TIMaE and TIME drives the discovery of dynamic biomarkers that are crucial for early detection. These biomarkers, including inflammatory cytokines, chemokines, and growth factors, emerge from the inflammatory processes inherent in tumor progression. Understanding the intricate TIME and TIMaE cross-talk is paramount in PC. Integrative approaches are therefore needed to facilitate better tumor characterization on both local and systemic levels as well as increase the possibility to identify the mechanisms underlying PC onset. This integrated analysis may assist in blood biomarker's discovery as a product of bidirectional communication between TME and the systemic component, providing important clinical diagnostic information in early PC lesions. To date, clinical trials are underway in order to validate these biomarkers and explore their potential in early detection, but continued research is warranted in order to refine diagnostic strategies, which will aid in the early identification of PC.

Based on the evidence we reviewed here it is time to take into consideration that cancer is a systemic disease that affects the body beyond the site of the primary tumor. Its systemic nature still requires comprehensive studies to intercept approaches for both diagnosis and treatment of PC in the next future.

## Data Availability

Not applicable.

## Abbreviations

PC: Pancreatic Cancer
PDAC: Pancreatic ductal adenocarcinoma
PanIN: Pancreatic intraepithelial neoplasms
IPMN: Intraductal papillary mucinous neoplasms
MCN: Mucinous cystic neoplasms
ADM: Acinar-ductal metaplasia
ECM: Extracellular matrix
TME: Tumour micro-environment
TiME: Tumour immune micro-environment
TIMaE: Tumor immune macro-environment
MDSCs: Myeloid-derived suppressor cells
TAMs: Tumor associate macrophages
BM: Bone marrow
HSCs: Hematopoietic stem cells
BMSCs: Bone marrow stem cells
MSCs: Mesenchymal stem cells
VSELs: Very small embryonic stem cells
BM-EPCs: Bone marrow-derived endothelial progenitor cells
PMN: Polymorphonuclear cells
PMN-MDSCs: Polymorphonuclear MDSCs
M-MDSCs: Monocytic MDSCs
GM-CSF: Granulocyte-macrophage colony-stimulating factor
KC: K-rasLSL.G12D/+; Pdx-1-Cre
KPC: K-rasLSL.G12D/+; Trp53R172H/+; Pdx-1-Cre
PSCs: Pancreatic stellate cells
TIMPs: Tissue inhibitors of metalloproteinases
α-SMA: α-Smooth muscle actin
CAFs: Cancer associated Fibroblast
myCAFs: Myofibroblastic CAFs
iCAFs: Inflammatory CAFs
apCAFs: Antigen-presenting CAFs
meCAFs: Activated metabolic state CAFs
CTCs: Circulating tumor cells
CA 19-9: Carbohydrate antigen 19-9
CEA: Carcino-embryonic antigen
CA125: Carbohydrate antigen 125
CA242: Carbohydrate antigen 242
MIC-1: Macrophage inhibitory cytokine-1
MUC5AC: Mucin 5AC
cfDNA: Cell free DNA
ctDNA: Circulating tumor DNA
NGS: Next generation sequencing
ncRNAs: Noncoding RNAs
EVs: Extracellular vesicles
GPC1+: Glypican-1
MIF: Migration inhibitory factor
AREG: Amphiregulin
